# In-depth data on the network structure and hourly activity of the Central Chilean power grid

**DOI:** 10.1038/sdata.2018.209

**Published:** 2018-10-23

**Authors:** Heetae Kim, David Olave-Rojas, Eduardo Álvarez-Miranda, Seung-Woo Son

**Affiliations:** 1 Department of Industrial Engineering, Universidad de Talca, Curicó 3341717, Chile; 2 Asia Pacific Center for Theoretical Physics, Pohang 37673, Korea; 3 Department of Applied Physics, Hanyang University, Ansan 15588, Korea; 4 Department of Physics and Astronomy, University of Calgary, Calgary, Alberta T2N 1N4, Canada

**Keywords:** Energy grids and networks, Complex networks

## Abstract

Network science enables us to improve the performance of complex systems such as traffic, communication, and power grids. To do so, it is necessary to use a well-constructed flawless network dataset associated with the system of interest. In this study, we present the dataset of the Chilean power grid. We harmonized data from three diverse sources to generate a unified dataset. Through an intensive review on the raw data, we filter out inconsistent errors and unrealistic faults, making the data more trustworthy. In contrast to other network dataset for power grids, we especially focus on preserving the physical structure of nodes’ connection incorporating the ‘tap’ structure. As a result, we provide three different versions of the dataset: ‘with-tap’, ‘without-tap’, and ‘reduced versions’. Along with structure, we incorporate various attributes of the nodes and edges such as the geo-coordinates, voltage of transmission lines, and the time series data of generation or consumption. These data are useful for network scientists to analyze the performance and dynamic stability of power grids.

## Background & Summary

With the recent development of network science, power grids have been studied to prevent large-scale blackouts, maintaining the stability of high-voltage transmission systems. For instance, studies on synchronization typically analyze the cascading consequences of malfunction in power-grid systems^1–7^. These studies rely on network datasets that embody the information of power grids. This information includes the connection topology of the transmission lines and the characteristics of each power facility: power plants or substations.

Usually, real-world power grids are mapped into network structures by performing aggregations and simplifications in order to produce convenient datasets for network analyses. As a matter of fact, in most cases such as the U.S.^7^ and EU countries including Italy^8^, France^8^, Spain^8^ and Great Britain^9,10^, the resulting datasets have only one type of transmission line and the amount of power consumption or generation is assumed to be uniform in all power-grid nodes. In terms of network terminology, those networks are unweighted and undirected, which are favorable only for analyzing general characteristics in the average sense.

Nowadays, power systems are comprised by a diverse pool of generation and transmission technologies, which respond to different operation schemes and to much more complex demand and revenue patterns. Therefore, it is important to use rather realistic network dataset in order to analyze more sophisticated (or realistic) operation issues on the power grids. Such a realistic network datasets should keep the detailed characteristics of power components such as capacity of each transmission lines (weight of edges), the amount of electricity consumption or generation of demand and power nodes, respectively, and geological coordinates (attributes of nodes). The attributes of network components will enable us to investigate the stability on the new-type of power grids, such as smart grid system, distributed power generation, and renewable power generation technologies.

There exist various methods for mapping a given power grid into a network representation^11^; this allows to generate power-grid networks with different degrees of accuracy. This means that, according to the scope, the power-grid network can be more detailed and realistic. It is obvious that the more accurate and realistic the performed analyses are, the more detailed network modeling is required^7^.

Indeed, some projects and protocols aim at constructing well-digitalized power-grid information. For instance, the European Network of Transmission System Operators for Electricity (ENTSO-E) is a global project among thirty-five European countries devoted to collecting network data of Pan-European power grids^12^; likewise, the Spatial Optimization Model of the Electricity Sector (ELMOD) is a model to digitize power grid data of Europe developed in 2012^13^. ELMOD covers the entire European electricity market. Continuing the effort of ELMOD, the Institute for Economic Research and the Technical University of Berlin recently developed ELMOD-DE, which particularizes the German case^14^. ELMOD-DE data package includes detailed power-grid information of German power grid is opened to the public. Complementary examples correspond to Sci-grid^15^ and GridKit^16^, which are open-source tool kits for automatically extracting power-grid topology information from OpenStreetMap^17^. Despite of these efforts, most of the well-structured data are concentrated in Europe and North America.

With the aim of addressing this issue, in this study we provide a curated network dataset of a power grid from South America. In particular, we map the Chilean power grid into three different network versions. Each of them is prepared by adopting or omitting one of two conversion schemes—*tapping* and *reduction*. These versions range from the most simplified topology to the most realistic one, enabling the users to selectively use a proper version considering the purpose of their analyses.

**Figure 1:**
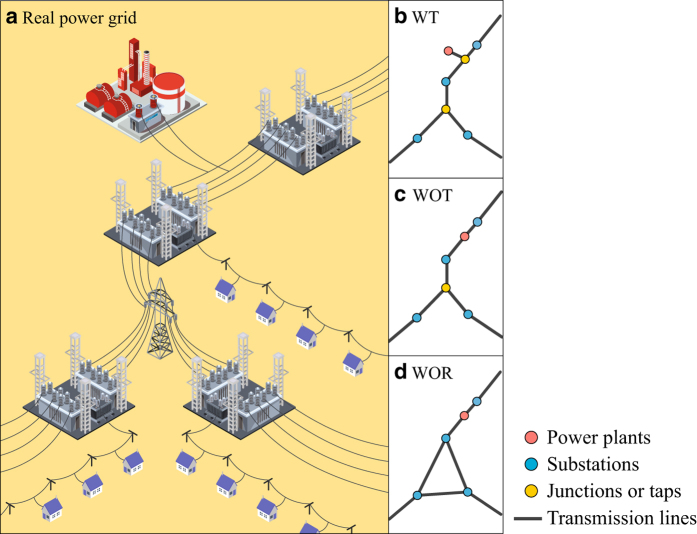
Three conversion schemes from a power grid to a network topology. A real power grid (**a**) can be represented in different versions. ‘With tap (WT)’ version in (**b**) has all power-grid components such as power plants, substations, taps, and junctions while ‘Without tap (WOT)’ version in (**c**) does not include taps. ‘Without tap reduced (WOR)’ version in (**d**) has the simplest structure having only power plants and substations.

In the first version, the one with the most realistic structure, power facilities are connected to the nearest transmission line by means of so-called tap connections. A tap connection is a short extension line from a facility to the main grid. Embedding this tap structure produces a complex connection structure, encompassed by four types of nodes with power plants, substations, taps, and junctions (Fig. 1b). This leads to a total of 347 nodes: 124 plants, 94 substations, 85 junctions (branch points), and 44 tap nodes. The second version, with intermediate degree of complexity, ignores these tap connections (Fig. 1c), and it encompasses 318 nodes: 124 power plants, 94 substations, and 100 junction nodes. Finally, the third, and most simplified version, aggregates nodes to a power plant and substation level, i.e., only *activity* nodes, which leads to a network comprised by 218 nodes (Fig. 1d). Besides the set of nodes, and the corresponding interconnection layout, we also provide the geo-coordinates and activity information of power-grid nodes that can be used for scenario-based analysis. This first, second, and third version of the networks will be referred to as *with-tap* network (WT), *without-tap* network (WOT), and *without-tap reduced* network (WOR).

## Methods

In Chile, the *Centro de Despacho Económico de Carga del Sistema Interconectado Central* (CDEC-SIC) is the agency devoted to the coordination and operation of the Central Chilean power grid. CDEC-SIC provides, in the website of Coordinador Eléctrico Nacional^18^, an extensive collection of raw data regarding the structure and operation of the generation and transmission network.

In particular, for this investigation, we merged three different sets of raw data into a unified dataset. Each source contains the geo-coordinates of power grid facilities (Mapa Sistemas Eléctricos de Chile^19^), the connection structure as a circuit diagram (Diagrama Unilineal SIC^20^), and the activity records regarding production and consumption (Operaciόn real^21^), respectively. In this section, we describe the detailed method used for the data cleaning process.

### Geographic coordinates

The data source containing geo-coordinates includes the longitude and latitude information of power plants and substations. The geo-reference system in the raw data is based on three time zones: EPSG 32717, 32718, and 32719, which correspond to UTM zone 17 S, 18 S, to 19 S. We convert the coordinate of 286 power plants, 860 substations, and 108 taps to EPSG 4326 system to have a standard geo-coordinate system.

In the process of integrating, cleaning, correcting, and processing the source data, we faced several difficulties due to imprecisions, lack of consistency, and missing information. In the following, we describe the main difficulties faced in the preparation process.

In the raw data, a relatively common situation corresponds to a mismatch among nodes’ ID and geo-coordinates when comparing different files. Hence, we have to individually inspect each problematic entry, in order to correct the geographical information, or ensure a correct association with respect to the node ID. Another troublesome situation occurs when mapping the connection layout associated with large substation complexes. For such situations, several transmission lines are connected to and from various directions, and each connection is at multiple locations within and around the complex. In this case, we merge substations into an *artificial* substation, whose location is given by the average value (center of mass) of the coordinates of the different substations comprising the complex.

Additionally, due to the fact that generators, substations, and other facilities belong to different owners, it is frequent that some of them have the same name. For instance, following the name of the area where the facility is located; e.g., two substations, owned by different operators, might be named *Curicó* because they are both located in a village named *Curicó*, and there could be more than one village with this name. Since part of the layout source data only associates names to facilities, having elements with replicated name produces a significant number of inconsistencies; most of them are manually corrected in a case-by-case analysis.

Furthermore, for some nodes of the power grid, the information regarding geo-coordinates is missing in the raw data. In these cases, we first manually checked each conflicting entry and attempted to assign coordinates by looking at the node ID, facility name, facility location, exiting connections, or any other relevant information at hand. Through this process, we corrected the information of 81 nodes in the case of WT (18.2% of the total nodes), and 59 nodes in the case of WOT (14.4%) and WOR (11.2%). When further manual assignment of coordinates was not possible, we indirectly set the locations by the following scheme. For the case of nodes with degree 1, we looked at the only connection of each of them, if the length of such connection is less or equal than a threshold value, then we set the missing node’s coordinates similar to its neighbor node. Complementary, for the case of nodes connected to several other nodes, we set the average value of the neighbors’ positions as the missing node’s geo-coordinates. These two steps are recursively applied to the data, until all nodes acquire geo-coordinates; the information of 59 nodes is corrected in the case of WT, and of 50 nodes in the case of WOT and WOR.

### Activity

For a given node, its *activity* corresponds to the amount of (net) power generation. If the node is a substation, the activity is non-positive; while if it is a generation node, the activity is non-negative. In particular, we collect the activity data for one year starting from 16th September 2015 until 15th September 2016, from the official web page of CDEC-SIC^18^. In order to minimize the human error during the data handling, we developed a Python code to automatically parse the data from the raw file.

Although all raw data are provided from the same organization, the information does not exactly coincide among the raw files. For example, ‘Sauzal’ is a unique node in the topology data. However, in the activity data, there exist ‘Sauazal 1’ and ‘Sauzal 2’. Because the activity data is made for the purpose of pricing and billing, each subunit is recorded individually. In this manner, we manually inspect the list of all raw data sources, and create a consolidated node list. In addition, for those nodes that do not have any activity data, we assume that the power facilities are not functioning due to maintenance. In that case, we set zero value for activity.

In reality the amount of power generation is always larger than the amount of consumption. The gap between the generation and the consumption is the so-called reserve capacity that compensates a sudden peak power demand preventing a blackout. In power studies, however, with the assumption of satisfying Kirchhoff’s law, it is common to set the net activity to zero. Therefore, by ensuring the grid to verify the Kirchhoff law, we are enabled to exploit electric principles associated with the grid. In this study, for the sake of applicability, we prepare two different versions of activity data: the original version and the Kirchhoff-adjusted version.

In the Kirchhoff-adjusted version, the net activity of each node is zero, i.e.,

(1)∑i=1Nai′=0,

where $a_{i}^{\prime }$ is the adjusted input or output of node *i* and *N* is the total number of nodes. In order to adjust the original values for making the total sum zero, we cancel out the reserve capacity from the generation activity. We do this by multiplying the activity values of generation with the ratio of the total consumption with respect to the total generation:

(2)ai′=rai,where{r=1ifi∈S,r=∑j∈Saj∑j∈Pajifi∈P.

Here *S* is the set of substation nodes, *P* is the set of power plant nodes, *a*_*i*_ is the original activity of node *i*, and $a{'}_{i}$Here *S* is the set of substation nodes, *P* is the set of power plant nodes, *a*_*i*_ is the original activity of node *i*, and $a_{i}^{\prime }$ is the adjusted activity of node *i*.

Since the product operation only affects to the nodes having non-zero activity value, the zero-activity nodes still remain inactive after the adjustment (See Equation (2)). This enables the adjustment process to still keep the unique characteristics of electricity generation profile of Chile.

### Connection structure

A power grid is a giant electrical circuit. In such circuits, power plants send electric power to substations through transmission lines. The single line diagram (SLD)^20^ of the Chilean power grid, which is one of the raw data sources, shows how the power plants and substations are connected.

The SLD is the ground truth of the Chilean power grid; based on it, we are able to construct the edge list between power-grid nodes. The SLD illustrates each power-grid component even for the individual generators in a power plant. The detailed information is useful to understand the connection layout structure of the power grid. For example, we can distinguish the capacity of transmission lines as 550 kilovolts (kV), 220 kV, 154 kV, 110 kV, 66 kV, and less than 66 kV, which can be used for the edge weight.

When mapping the SLD into a network structure, we apply, in particular, two conversion principles in this study: tap-embedding and node reduction. The practical conversion process is described in each subsection below.

### Tap-embedding and further network characterization

*Tapping* is a connecting method of a node to the middle of a transmission line. An extended line from a node hangs on a spanning transmission line, which enables the node to connect the main grid. The tapping has an advantage in system’s stability point of view. When an accident or a failure happens at the tapped node, it does not affect the current flow of the main grid. However, when the node is placed between two nodes without tapping, all three nodes are connected in order. In this case, a single failure on the middle node could directly break the current flowing through the node, which makes the grid vulnerable. The tapping connection is to preserve the connectivity between other nodes regardless of the functionality of the tapped node.

One can find tap structures often in real transmission grids. However, for the sake of simplicity, their structure is usually simplified when it is converted into a network topology. The topological structure is one of the main ingredients for the network analysis on power grids. Whether we ignore the tap or not, directly affects the result. Therefore, one should carefully decide to include or exclude the tap structure based on the purpose of the analysis. In order to characterize the tap connection, we keep all tapped structures from the SLD, which could be identified either by their name (explicitly identifying a tap role) or they connection arrangement.

Besides tap connections, *junction points* also matter from a structural point of view. At a junction point, transmission lines are merged (or diverged) not incorporating any power facility such as generators or substations. We consider the junctions as nodes when we find from the SLD of the Chilean power grid. This enables us to keep the characteristic of the power grid.

From the SLD, we classify the role of nodes from the corresponding symbol. The nodes with transformer symbols are substations; generator symbols, power plants. As a result, we collect 818 nodes from the SLD: 463 substations, 168 plants, 51 junctions, and 136 tap nodes.

Among the original 818 power-grid nodes, we only keep those appearing in all data sources. To do so, when we remove the nodes that exist only in some data sources, we keep the connectivity between the adjacent nodes making alternate edges to prevent network segregation. However, in order not to make any duplicated edge, we remove the multi-edges between the same nodes. The final network data include only the nodes commonly exist in all data sources: 347 nodes in tap-embedded version and 318 nodes in without-tap version.

### Node reduction

So far, we have explained two versions of the Chilean power grid: WT and WOT. In order to construct the most simplified version (WOR)—but most structurally similar to other power-grid dataset—we further reduce nodes, using the so-called star-mesh transformation principle^22^.

**Figure 2:**
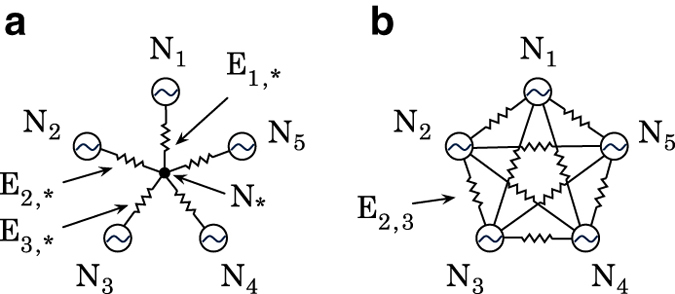
Star-mesh conversion results in the electrically equivalent circuit removing the center node. Conversion from (**a**) a star-shape circuit to (**b**) a mesh shape. *i* is the node index and ^∗^ represents the central node that is being removed by the transformation process.

Star-mesh transformation is a useful technique to convert a circuit to other electrically equivalent configuration with less number of nodes (See Fig. 2). However, the voltages applying to outer nodes are still identical for both configurations. Therefore, it is useful to simplify power grids keeping the electrical relationship between nodes. When the reduction process is conducted to only four nodes, the conversion process is called Kron reduction^23–25^ or Y-Δ transformation.

This transformation implies that edge characteristics shall be mapped from the information of the original setting. For the case of power grids, the resulting edges impedance is based on the impedance of edges connecting the central node to the peripheral nodes, according to

(3)Ei,j=Ei,*Ej,*∑i∈n.n(*)1Ei,*

where ^∗^ is the index of the central node, *i*, *j* are the node indices connected to the central node, and *E*_*i,j*_ corresponds to the impedance of the transmission line between node *i* and *j*. Applying the Star-mash transformation to WOT version, we construct WOR version of dataset with total 218 nodes.

### Code availability

We used command line tools to avoid any potential human error during manual work. All library and modules used in this study are freely available open access tools. During the data process, we mainly used *Python 2.7* along with *Numpy 1.9.1* and *Pandas 0.19.2* for parsing and cleaning data. To visualize the network we used *Basemap 1.0.7* and *Matplotlib 1.4.2*.

## Data Records

**Figure 3:**
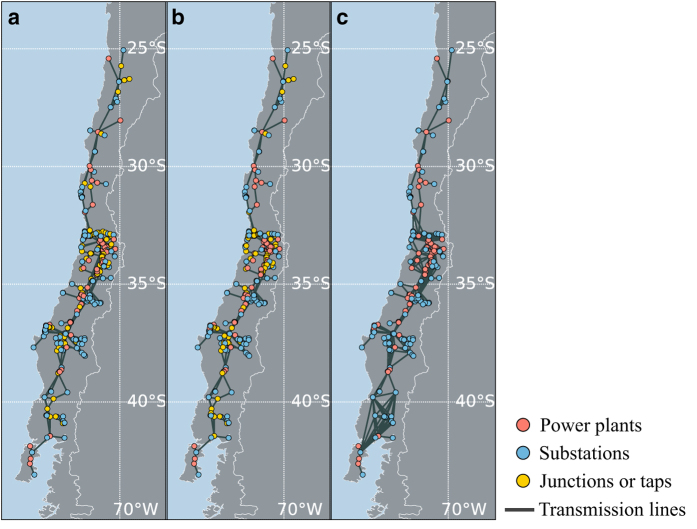
Network representation of Chilean power grid on the map of Chile. The network components in Chilean power grid are converted according to the concept of (**a**) WT, (**b**) WOT, or (**c**) WOR, respectively. Nodes are located on the actual geo-coordinates such that some nearby nodes are illustrated overlapping.

We prepared three versions of power-grid dataset by selectively combining different conversion techniques that are described before. These versions are shown in Fig. 3a–c, corresponding to WT, WOT, and WOR, respectively, i.e., ranging from a connection structure including all tap and junction points, to a structure including only activity nodes.

All versions of the network preserve the functional relationship between nodes in terms of dynamics. The brief information regarding the three versions is in Table 1.

The network dataset are prepared in a data repository (Data Citation 1). Files are organized by versions having the name of each version in the front of file name. The information with respect to nodes, edges, and activity are prepared as *.csv* files and the name of the file tells what it includes. In the node list, each row corresponds to a node and columns show the detail attributes of the node. To clarify the data, the node attributes include following information:

- ID: the identification index of the node that is also used in the edge list
- Label: the name of the node
- Owner: the company that owns the power facility
- Role: the kind of the node (power plant, substation, tap, or junction)
- Longitude: the longitude of the node
- Latitude: the latitude of the node

The edge list has the detailed transmission connection status of the power grid. ‘Source’ and ‘Target’ columns in the file mean end points of an edge. Note that all edges are indirect connections. Edge information in the file includes:

- Source: the ID of the end point of an edge
- Target: the ID of another end point of the edge
- Distance: the distance of the corresponding transmission line (km)
- Voltage: the capacity of the transmission line (kV)

Activity data is encoded into a large spreadsheet with time series information about the amount of consumption or generation (MWh) of activity nodes. For users’ convenience, we also leave the node attributes in the same file. The file includes 8788 rows in total: 4 rows for role, ID, label, and owner of a node plus 8784 rows for time stamp from 16th September 2015 to 15th September 2016 (366 days) on an hourly basis. Note that within the period, there is no importation nor exportation of electricity associated with SIC. The central Chilean power grid is self-sustaining independently to neighborhood as a closed system.

### Power grid with tap

As explained above, the tap-embedded network data reflects the physical connection structure of power grids. We leave out tap connections and junctions as they exist as a part of the real power-grid structure. This version is particularly useful to simulate the network response against physical attacks. In this version of the data, the network is comprised by 444 edges, and by a total 347 nodes consisting of 124 plants, 94 substations, 85 junctions (branch points), and 44 tap nodes.

### Power grid without tap

In this version power plants and substations are no longer connected via taps. The tap nodes are removed except for the case that they are necessary from the structural point of view. For the exceptional cases, we set the attribute of the tap nodes as junctions since they are making branches. The network topology in this version still reveals the unique physical structure of the power grid; it is the intermediate version between the most realistic tap-embedded version and the most simplified Star-mesh reduced one. In this case, there are 409 edges and there exist 318 nodes in total (124 power plants, 94 substations, and 100 junction nodes without any tap node).

### Reduced power grid

The most simplified network form of power grid is the reduced network. It eliminates the redundant nodes that do not affect the dynamical interaction between power producers and consumers. For instance, network studies about synchronization problems usually consider only power producers and consumers as oscillators. In this case, junctions and taps are ignored because they do not affect the dynamic interaction between the oscillators. Most network studies utilize power-grid networks that contain only plant and substations, the reduced version is useful especially to compare with other studies. This reduced version has solely 527 edges with 124 power plant nodes and 94 substations.

### Activity data

The total power production for an year is about 47,005 GWh in the original data with 3,176 GWh of reserve capacity that is in good agreement with the normal 10–20 percent of reserve ratio. In the Kirchhoff-adjusted dataset, the total power generation is scaled to be equal to the total amount of power consumption, which results in 43,829 GWh of power production. The maximum power producer during the period is *Guacolda* with 4,499 GWh (4,195 GWh after scaling) of power generation and the maximum consumer is *Polpaico* with 3,054 GWh.

**Figure 4:**
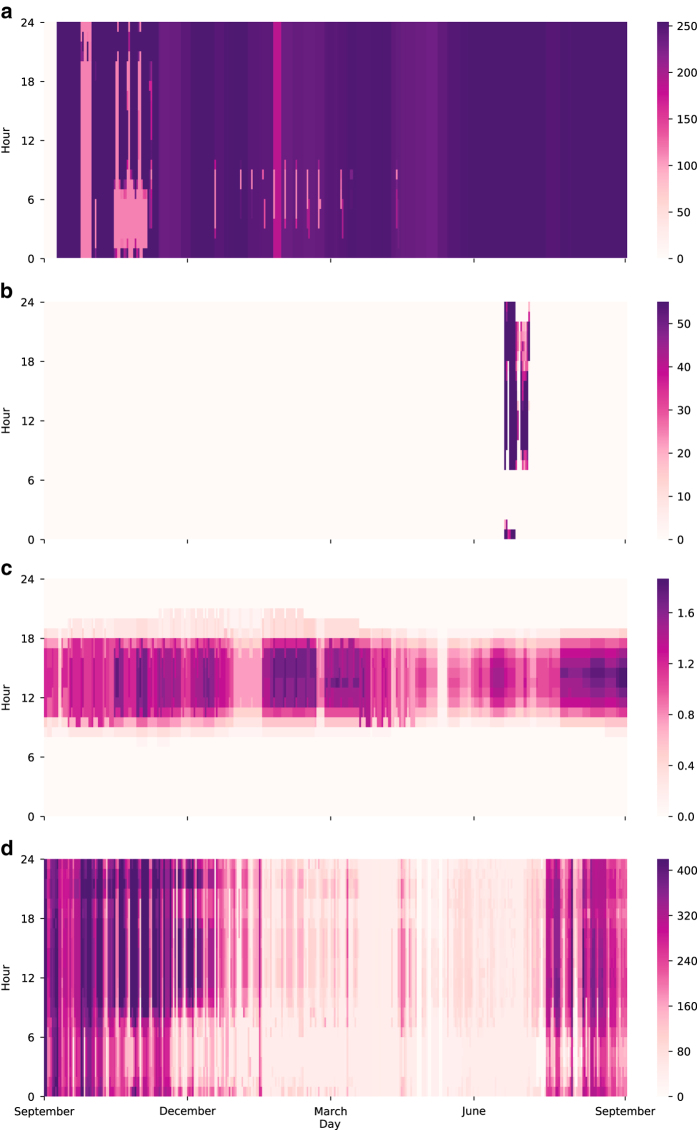
Hourly activity record of few power plants in Chile from 16th September in 2016 to 15th September in 2017. The power plants show various generation patterns according to the generation technology of (**a**) Campiche, (**b**) Teno, (**c**) Loma Las Colorados, and (**d**) Pangue (in the unit of MWh).

Activity data, which is associated to generators and sub-stations, is useful to analyze power grids considering the actual demand and supply pattern of consumers and power plants, respectively. Figure 4 shows heat maps of activity data of four power plants in the Chilean power grid. The activity data clearly shows the distinct pattern of power generation according to the season, time, and technology. It enables us to do a scenario-based case study with historical records.

For example, Campiche (Fig. 4a) is one of the base power facilities in Chile and constantly supplies electric power. On the other hand Teno (Fig. 4b) generates electricity during only a specific period. The activity pattern also reveals the technological and seasonal characteristics of power activity of nodes. Loma Las Colorados (Fig. 4c) is a solar power plant such that it generates only during day time. The electric power generation of hydro power plant increases during the rainy season as Pangue shows in Fig. 4d.

## Technical Validation

As the constructed network corresponds to a unified interconnected power system, all nodes shall comprise a single large connected component. Therefore, in the three data versions we ensure that connectivity was verified by the resulting network, through a depth-first search algorithm.

**Figure 5:**
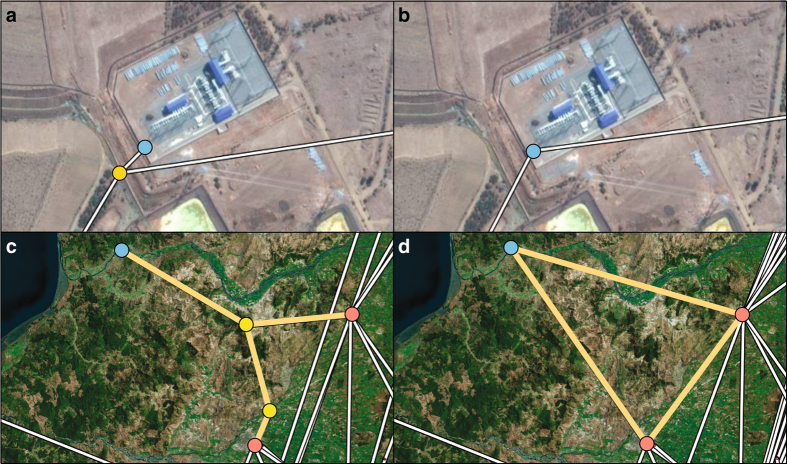
Visual comparison between WT, WOT, and WOR versions. The different structure between WT (**a**) and WOT (**b**) shows the role of tap connection. The tap node (yellow) in WT (**a**) is the tapping point of the power plant, *Lo Aquirre* (blue) to the transmission line. The redundant junction nodes (yellow) in WT (**c**) are eliminated in WOR (**d**). The edge connection also changes (yellow lines) in order to generate electrically equivalent circuit.

While the connectivity can be checked by a standard procedure, the remaining characteristics can be only verified by an exhaustive brute-force method. For instance, the correct removal of a tap or junction node, can be only verified by analyzing candidates one by one. A tap node (yellow) in Fig. 5a, for example, is correctly eliminated after applying our conversion procedure resulting in a reduced network Fig. 5b. A redundant central node (yellow) in Fig. 5c, along with its connected edges, is also removed in the reduced version in Fig. 5d. Note that the resulting networks shown in Fig. 5a and c correspond to WT, Fig. 5b to WOT, and Fig. 5d to WOR, respectively. We also check that the sum of activity of all nodes in Kirchhoff-adjusted versions is less than −3.72×10^−7^.

## Usage Notes

One of the easiest ways to use the activity data provided by this study is to import with the *Pandas* library in *Python*. For the sake of users’ convenience, we include the attribute of nodes–ID, name, and owner–as the multiple indexes in *.csv* file along with the activity data. Since the activity data is prepared by using standard packages and recorded with UTF-8 text encoding, it is available in any programming language without sensitive development environment issues.

Here we describe usage examples of our dataset. For those who want to use this data for the purpose of comparison to other power grids, it is recommended to use the reduced version. It is because of the fact that most current network dataset of power grids in many studies contain only power plant and substation nodes^2,6,26–30^. The reduced version is the general type of power-grid data at the moment.

On the other hand, when one needs a detailed structure of power grids for analyses related with the physical connection topology or optimal power flow^30–33^, the ‘with-tap’ or ‘without-tap’ version is a good choice. The ‘with-tap’ version is a dedicated version for those who are interested in zooming in the networks. The ‘without-tap’ version ignores tapping but still preserves the overall topological shape such that it is appropriate for the analysis in which the difference due to tapping is negligible.

## Additional information

**How to cite this article**: Kim, H. *et al.* In-depth data on the network structure and hourly activity of the Central Chilean power grid. *Sci. Data*. 5:180209 doi: 10.1038/sdata.2018.209 (2018).

**Publisher’s note**: Springer Nature remains neutral with regard to jurisdictional claims in published maps and institutional affiliations.

## Supplementary Material



## Figures and Tables

**Table 1:** The number of nodes and edges in each version.

	**Node**					**Edge**
	**Power plant**	**Substation**	**Tap**	**Junction**	**Total**	
WT	124	94	44	85	347	444
WOT	124	94	0	100	318	409
WOR	124	94	0	0	218	527
